# Oral Ciprofloxacin Prophylaxis in Patients Undergoing High DoseTherapy and Autologous Hematopoietic Stem Cell Transplantation

**Published:** 2016

**Authors:** Mahdi Tabarraee, Maria Tavakoli-Ardakani, Mahshid Mehdizadeh, Mojtaba Ghadiani, Hamid Rezvani, Abbas Hajifathali, Samiyeh khamsi

**Affiliations:** a *Hematology and oncology center, Taleghani Hospital, Shahid Beheshti University of Medical Sciences, Tehran, Iran.*; b *Department of Clinical Pharmacy, School of Pharmacy and Pharmaceutical Sciences Research Center, Shahid Beheshti University of Medical Sciences, Tehran, Iran. *; c *Taleghani Bone Marrow Transplantation Center, Taleghani Hospital, Shahid Beheshti University Of Medical Sciences, Tehran, Iran. *; d *Islamic Azad University in Medical Sciences, Tehran, Iran.*

**Keywords:** Oral Ciprofloxacin, Prophylaxis, Autologous Hematopoietic Stem Cell Transplantation, High dose chemotherapy

## Abstract

Antibiotic prophylaxis is usually used in allogeneic stem cell transplantation, but its use in Autologous Stem Cell Transplantation (ASCT) is controversial. We evaluated the efficacy of ciprofloxacin prophylaxis in ASCT.

To identify the efficacy of ciprofloxacin on the incidence of neutropenic fever and its complications, 72 patients that had been admitted to Taleghani Hospital for ASCT between 2010 and 2012 were evaluated in our study. Oral ciprofloxacin 500 mg every 12 h was administered to 30 patients on the same day of high dose chemotherapy until the first febrile episode or until the recovery of neutropenia and the results were analyzed and compared with the historical control group 42 other transplanted patients who had not previously received ciprofloxacin.

The incidence of neutropenic fever was 80% with no difference between the two groups. But in ciprofloxacin group, duration of fever (1.7 days VS 3.5 days P=0.017), hospitalization due to stem cell transfusion (18.2 days VS 12.2 days p=0.03), incidence of bacteremia 3.3 % VS 33.3%, p=0.002) and platelet recovery (13.9 VS 17.7 days= 0.035) and platelet transfusions (P=0.04) were significantly lower than the control group no side effects and no delay in.

Based on this study oral ciprofloxacin prophylaxis is rational, efficacious and economic in ASCT.

## Introduction

Infections complications are the major causes of morbidity and mortality in hematopoietic stem cell transplantation (HSCT) (1).

Even though the risk of death due to infection is much lower after autologous transplantation (compared to allogeneic transplantation). The risks of procedure are greater than those of conventional chemotherapy, and preventive policies should be implemented in any transplant program. Because of the hospital environment and bacterial resistance, the physical environment of transplant patients should aim to decrease the risk of nosocomial infection (2).

The gut is the main reservoir for gram-negative bacteria and a source of subsequent entry into the body. Gut decontamination is approached in different centers and countries. The fluoroquinolones, introduced in the 1980s, have transformed this field, becoming the most commonly used prophylactic antibacterial agents in neutropenic patients because of their broad antimicrobial spectrum, preservation of anaerobic gut flora, systemic bactericidal activity, good tolerability and lack of myelosuppression. In acute leukemia patients, a large trial comparing levofloxacin to placebo has shown that the use of levofloxacin during neutropenic phase significantly reduces the risk of fever, bacterial infection, bacteremia, especially of gram-negative infections, and cost of intravenous antibacterial medicine for febrile neutropenia (3, 4,5,6,7 and 8). The medicine should be given until the first febrile episode or the recovery of neutropenia in the absence of fever. There is no doubt that routine prophylactic use of antibiotics can cause colonization of an individual patient with resistant organisms; but, the clinical relevance of this is unclear. Before 2005, few trials had been performed and none were large enough to provide conclusive evidence on the benefit of prophylaxis (9, 10, 11, 12 and 13). Bucaneve *et al*. (2005) observed a non-significant increase in the incidence of levofloxacin-resistant gram-negative bacteremia among patients receiving levofloxacin, but this did not affect outcomes such as infection-related morbidity or mortality. Therefore, their use is not recommended in units with a high level of quinolone resistance among gram negative bacteria and should be associated with periodic monitoring of local epidemiology as well. (14).

## Experimental

 In this study in Taleghani hematopoietic stem cell transplantation center, 90 patients who had undergone ASCT from January 2010 to December 2011 as treatment for multiple myeloma, malignant lymphoma (Hodgkin's disease and non-Hodgkin's lymphoma), AML or solid tumors were eligible to enter the study. Patients younger than sixteen years old, any active infection, detection of pathogenic organism in specimen cultures or antibiotic usage on admission were excluded from the study. Hospital records of ninety patients were evaluated, eighteen patients had excluding criteria and 72 patients enrolled to our study. Patients in group A received prophylactic oral ciprofloxacin, 500 mg every 12 h, from the first day of high dose chemotherapy until the first day of febrile episode or engraftment. Patients in group B did not receive prophylactic antibiotic. Patients in both groups received prophylactic acyclovir 400 mg/BID and fluconazole 400 mg/daily. Cytomegalovirus antigen (CMVpp 65Ag) was measured weekly. In this study 30 patients whom received ciprofloxacin entering group A and 42 who did not receive ciprofloxacin entering the historical control group. The least samples size in each group was calculated with following formula α = %5, B=%10, P_1_=%50, P_2_=%90

$n=\frac{{\left(Z_{1}-\alpha /2+Z_{1}-B\right)}^{2}[P_{1}\left(1-P_{1}\right)+P_{2}(1-P_{2}}{{\left(P_{1}-P_{2}\right)}^{2}}$

$23=\frac{{\left(\frac{1}{96}+\frac{1}{28}\right)}^{2}[0.5\times 0.9\times 0.1]}{{\left(0.5-0.9\right)}^{2}}$

All patients received GCSF only protocol for stem cell mobilization. Patients who received allogeneic stem cell graft excluded from this study. Written informed consent was taken from all patients. All patients had general health criteria for (HSCT) (by cardiologist, psychologist, and dentist consultation). Hematopoietic stem cell transplantation was carried out according to standard protocols .Microbial cultures from urine, blood, different parts of skin and throat were performed for all patients. Pretransplant conditioning based on type and phase of disease and included the high dose chemotherapy followed by reinfusion of previously harvested autologous peripheral blood stem cells. Patients were physically examined for infectious signs every day and the temperature was routinely registered every 6 h routinely. Fever was defined with an episode of oral temperature equal or over 38.3 C° or 38 continuing for at least one hour.

*Statistical analysis*

Continuous variables were expressed as mean value ± standard deviation. Data analysis was performed using chi-square test, Fisher's exact tent, unpaired t test, and Mann Whitney U test. A p-value less than 0.05 were considered to be statistically significant. The statistical analysis was performed with SPSS vs. 17.0.

## Results

From January 2010 to December 2011, 72 patients were evaluated, with 30 patients assigning into the prophylaxis group and 42 assigned to the control group. There were no significant differences between the two study groups regarding to the demographic and other characteristics (Table 1).

**Table 1:** Characteristics of patients in two groups.

**Patient characteristics**	**Group A**	**Group B**	**P value**
**Age**	37.4 (22-52)	39.74 (25-53)	
**Gender **			
Male Female	19 (63.3%) 11 (36.7%)	27 (64.3%) 15 (35.7%)	1.000 1.000
**Disease**			
NHL^a^ HD^b^ MM^c^	6 (20%) 12 (40%) 10 (33.3%)	8 (19.5%) 17 (41.5%) 15 (36.6%)	0.850 0.852 0.811

a NHL: Non Hodgkin Lymphoma

b HD: Hodgkin Disease

c MM: Multiple Myeloma

The incidence of neutropenic fever and duration of neutropenia were similar in both groups (Table 2). But there was a significant difference in the total number of febrile days and length of hospital stay after transplantation in prophylaxis and control groups (Table 2). There was no significant difference in neutrophil engraftment between the prophylaxis and control groups but, a statistically significant difference in the day of platelet engraftment and the number of platelet transfusions was detected between the two groups (Table 2). Positive blood cultures were significantly more in the control group (Table 2).

**Table 2:** Outcomes of patients i.

**Outcomes** **of patients**	**Group A**	**Group B**	**P value**
Neutropenic Fever	24 (80.0%)	35 (83.3%)	0.763
Positive culture	1 (3.3%)	14 (33.3%)	0.002
Days of ANC<500	8.13% (3-12)	8.6 (3-13)	0.692
Day of ANC.ENG	12.9 (8-15)	13.2 (8-18)	0.798
Days PLT<20000	6.6% (1-11)	10.3 (1-20)	0.047
Day of PLT.ENG	13.9 (9-18)	17.7 (8-27)	0.035
No of Blood Transfusion	1.1 (0-2)	2 (0-5)	0.077
No of PLT Transfusion	1.7 (0-3)	2.8 (0-5)	0.041
Duration of Admission	18.2 (13-23)	22.2 (13-31)	0.032
Fever .Duration	1.7 (1-3)	3.5 (1-8)	0.017
Int.N-F	3 (1-5)	2.7 (1-5)	0.557
Fever Day	6.0 (5-11)	5.5 (3-9)	0.492

ANC: Absolute Neutrophil Count, ENG = EngrftmentInt, N-F =Interval between Neutropenia & Fever

Fever Day = the day fever begins

## Discussion

Several randomized studies evaluated effects of quinolones prophylaxis on the rates of fever and infection in cancer patient with chemotherapy-induced neutropenia [3, 4, 5, 15, and 16]. Other studies evaluated effects on mortality rate (17, 18, and 19).

In one large study by Bucaneve *et al*. (2005) 760 hospitalized adult patients with acute leukemia, lymphoma, or solid tumors whose chemotherapy induced prolonged neutropenia (longer than 7 days) were randomized to receive either oral levofloxacin 500 mg daily or placebo from the start of chemotherapy until the resolution of neutropenia (3). This study and other two studies by Bucaneve *et al.* and Cullen M, *et* *al*. have documented the beneficial effects of levofloxacin prophylaxis on reducing rates of fever and infection in cancer patients with neutropenia (14,19). In two different studies by Gafter-Gvili A, *et al. *and Leibovici *et al*., it was shown that mortality reduced by antibiotic prophylaxis in neutropenia patients following chemotherapy (18, 20)

In one study by KA Guthrie *et al* during allogeneic hematopoietic cell transplantation, levofloxacin accompanied lower rates of bacteremia than ceftazidime (day 100, 19.2 VS 29.6%, P=0.02)

Many studies have documented usefulness of quinolone prophylaxis in reducing rates of fever and infection in cancer patients with neutropenia and during allogeneic bone marrow transplantation (21). In a meta-analysis of randomized, blinded, placebo-controlled trials by Imran H, Tleyjeh IM, a total of 2,721 patients with solid and hematologic malignancies were randomized in eight eligible trials (22). Comparing with the placebo, there was a statistically non-significant but consistent decrease in mortality with fluoroquinolone prophylaxis (4.5% vs. 3.9%, relative risk (RR) 0.76, 95% confidence interval (CI) 0.54, 1.08, p = 0.13, I (2) = 0%). 

In our study we evaluated beneficial effects of ciprofloxacin during autologous bone marrow transplantation. Although the incidence of neutropenic fever was similar in control and ciprofloxacin groups (83% VS 80%), but duration of fever (1.7 days VS 3.5 days P=0.017) and hospital stay from stem cell transfusion (18.2 VS 22.2 P=0.03) were shorter in the ciprofloxacin group than the control group. This means that severity of infection is lower in ciprofloxacin group. Although some investigators have advocated caution for antibiotic prophylaxis because to possible increase in enteric infections such as C.difficile (23, 24), our study shows these infections are is not considerable and cannot increase duration of hospitalization.

In addition, the incidence of bacteremia and the number of platelet transfusion were lower in ciprofloxacin group, that may be related to bone marrow suppression and peripheral consumption during infections disease .Although the beneficial effects of ciprofloxacin were shown during high dose chemotherapy and autologous bone marrow transplantation, but studies should be repeated periodically to evaluate the patterns of pathogens and resistance in any patient population and assess the effectiveness of antibiotic prophylaxis .

## Conclusion

There is now convincing evidence that antibiotic prophylaxis reduces duration of fever and neutropenia and duration of hospitalization in patients with lymphoma, multiple myeloma, and solid tumors receiving high-dose chemotherapy in HSCT setting. Therefore, we recommend routine antibiotic prophylaxis in these groups of patients. Fluorquinolones are effective and well tolerated for prophylaxis. Among the quinolones, we should take the patterns of pathogens and resistance in our patient population into account. Therefore based on this study using of oral quinolones (ciprofloxacin) for prophylaxis may be rational, efficacious, and economic in ASCT.
